# Fifteen Years of Patient Experience with Hospital Food in a Spanish Long-Term Care Hospital

**DOI:** 10.3390/nu18081246

**Published:** 2026-04-15

**Authors:** M.ª Isabel Ferrero-López, Clara Pérez-Esteve, Mercedes Guilabert Mora, Cristina M.ª Nebot-Marzal, José Mira

**Affiliations:** 1 Hospital Padre Jofre, 46017 Valencia, Spain; ferrero_isa@gva.es (M.I.F.-L.); nebot_cri@gva.es (C.M.N.-M.); 2 Foundation for the Promotion of Health and Biomedical Research of the Valencian Region (FISABIO), 03010 Alicante, Spain; clara.pereze@umh.es (C.P.-E.); jose.mira@umh.es (J.M.); 3 Departamento Psicología de la Salud, Universidad Miguel Hernández, 03202 Elche, Spain

**Keywords:** hospital food service (MeSH), patient satisfaction (MeSH), quality improvement (MeSH), long-term care (MeSH)

## Abstract

**Background/Objectives**: Adequate nutrition in older adults is essential to maintaining health, functionality, and quality of life, particularly in long-term care hospitals (HACLEs). Previous studies suggest that dissatisfaction with hospital food is linked to longer stays, more complications, and negative perceptions of care. Given these concerns, this study aimed to assess patients’ experiences with hospital food over a 15-year period in a HACLE in Spain, identify key influencing factors, and validate an updated PREM (Patient Reported Experience Measure) tool for food services. **Methods**: A retrospective, observational, repeated cross-sectional study was conducted using annual PREM surveys administered between 2011 and 2025 to patients on oral diets. Psychometric validation of the updated 8-item version (2024) was conducted. **Results**: Out of 1618 surveys, 1540 were included in the final analysis. The updated PREM showed strong internal consistency (α = 0.85, ω = 0.87), a two-factor structure (food quality and service conditions), and adequate model fit. Perceptions worsened after a catering company change in 2022 but improved following the implementation of new food distribution carts in 2025. The PREM total score showed a strong positive association with the global satisfaction item, providing supportive evidence based on a closely related anchor measure (Spearman’s rho = 0.80, 95% CI 0.77–0.82; *p* < 0.001). Scores differed significantly by diet type: patients receiving a pureed diet reported the highest average satisfaction score, followed by those on a soft diet and a regular diet. The group on a soft diet excluding foods that pose a choking hazard had the lowest mean score. **Conclusions**: The validated PREM scale is a reliable tool to monitor patient experience with hospital food. It enables early detection of quality issues and supports targeted improvements. Routine use in long-term care settings may foster personalized, patient-centered nutrition strategies and enhance care quality.

## 1. Introduction

Adequate nutrition in older adults is essential for maintaining health, functionality, and quality of life, particularly in long-term care hospitals where this population is predominant [1]. Nutritional status plays a key role in all healthcare settings, especially during illness and hospitalization. The PREDYCES study showed that approximately one in four hospitalized patients is malnourished, and that some patients even develop malnutrition during their hospital stay [2]. Disease-related malnutrition (DRM) is a multifactorial condition, frequently associated with acute or chronic inflammation, reduced food intake, malabsorption, or increased metabolic demands due to the underlying disease [3].

Malnutrition has important clinical, functional, and economic consequences. It is associated with increased risk of complications, infections, and mortality, delayed recovery, prolonged hospital stays, higher readmission rates, and increased healthcare costs. In addition, it contributes to muscle loss, sarcopenia, reduced autonomy, and poorer quality of life [4]. Among the identified causes of malnutrition is the quality and appearance of the food served during hospitalization [5].

A negative assessment of hospital food often precedes an overall negative perception of care received, longer lengths of stay, and an increased number of complications [6,7,8].

Most studies examining patient evaluations of hospital food services have been conducted in the United States, the United Kingdom, and a few European countries [7]. These studies typically rely on questionnaires—many of them not validated—that tend to report generally positive evaluations of hospital food. Key factors contributing to a positive experience include food quality, presentation, and the overall service provided [1].

In Spain, long-term and chronic care hospitals (HACLEs) were established in the mid-1990s to optimize hospital resources, with a focus on complex chronic patients, individuals with disabilities, and those requiring prolonged medical treatment [9]. Proper nutrition is a critical component in the effectiveness of these institutions. This study was designed to assess patient experiences with food during their stay in a long-term care hospital, to identify key factors influencing these perceptions over time, and to update an existing Patient-Reported Experience Measure (PREM) by analyzing its psychometric properties.

## 2. Materials and Methods

This was a retrospective, observational, repeated cross-sectional study based on secondary analysis of data systematically collected through a nutrition-focused Patient-Reported Experience Measure (PREM) administered to hospitalized patients at a long-term care hospital (HACLE) in the Valencian Community between 2011 and 2025.

To strengthen the interpretation of findings, the manuscript was organized into two complementary studies. Study 1 focuses on the metric properties of the PREM used in this setting, assessing its validity and reliability. Study 2 presents the results obtained with this instrument across the analyzed period, with particular attention to temporal trends and differences according to relevant clinical and organizational factors.

### 2.1. Setting

The HACLE has a total of 125 beds, of which 50 are allocated to mental health care and 75 to high-risk, frail older adults.

### 2.2. Data Source

Data were obtained from institutional records maintained by the HACLE. The datasets were curated to ensure data quality and consistency.

### 2.3. Nutrition PREM

The instrument used at the HACLE since 2011 initially consisted of nine items and one item of overall opinion of the food service, rated on a 4-point Likert scale (0 = Poor to 3 = Very good) (Supplementary Material Questionnaire S1). In 2023, a review of existing patient experience questionnaires on hospital food services was conducted to inform item development. Drawing on the examiners’ prior experience, a working group was established to draft a set of questions addressing various aspects of the food service. The resulting PREM consisted of eight items and one item of overall opinion of the food service, rated on a 5-point Likert scale (0 = Poor to 4 = Very good), covering key elements of the food experience. The transformation from a 4-point to a 5-point Likert scale was implemented to increase response variability and improve discriminative capacity, including a neutral midpoint to reduce forced-choice bias. As part of this revision process, the item assessing portion size (“How do you consider the quantity of the food?”) was removed, following validation analyses that revealed low correlation with the other items and limited contribution to the overall construct of perceived quality. This version was subsequently used in 2024 and 2025 (Supplementary Material Questionnaire S2).

### 2.4. Participants

Given an average of approximately 450 admissions per year, all patients receiving an oral diet and present at each annual assessment point were eligible and invited to participate. When patients were unable to complete the questionnaire themselves, a family member or paid caregiver responded on their behalf, provided they were directly involved in the patient’s daily feeding routine or meal-related care.

### 2.5. Procedure

The PREM was administered on paper once per year, typically in May, to all inpatients on oral diets. Nurses from each ward distributed the questionnaires, and the process was coordinated by the hospital’s Nutrition and Dietetics Unit. Each response was linked to the type of diet prescribed. Each annual administration was treated as an independent cross-sectional snapshot, and analyses were conducted assuming independence of observations across years.

### 2.6. Ethical Considerations

The study was approved by the Research Ethics Committee for medicinal products (CEIm) of Hospital Doctor Peset in Valencia (Approval Code: 040/25). Data protection in accordance with applicable regulations for each year of data collection was ensured by the HACLE itself, including strict confidentiality through data anonymization.

### 2.7. Study 1. Psychometric Properties of the PREM

In 2024, based on the analysis of historical data, an internal consistency review led to the reduction of the instrument by eliminating one item due to its low item-total correlation (r = 0.16). Additionally, the working group decided to adopt a 5-point Likert scale to increase response variability and allow participants to express their agreement or disagreement with greater precision, addressing the positive skew observed in previous years (Supplementary Material Questionnaire S2).

To harmonize responses from earlier years with the new version, a data transformation was applied to align all responses on a common scale (assigning a value of 1 to former “0” responses), allowing for consistent comparison across different Likert scale versions. This updated 8-item version was assessed following COSMIN guidelines, considering the following aspects:

**Face and Content Validity:** The content and wording of the questions were developed and refined by the staff of the hospital’s nutrition department, who ensured the relevance and appropriateness of each item. Psychologists with expertise in geriatric care reviewed the clarity, comprehension, and ease of response to assess understanding, response burden, and the face validity of the instrument.

**Item Analysis:** Each item’s item-total correlation and discriminatory power were examined.

**Structural Validity:** An exploratory factor analysis (EFA) was first conducted to determine the empirical structure. The Kaiser-Meyer-Olkin (KMO) measure and Bartlett’s test of sphericity were used to assess factorability. Principal component analysis with oblimin rotation was applied, with factor loadings ≥0.30 considered meaningful. Confirmatory factor analysis (CFA) was then used to test the proposed structure, using fit indices such as the Comparative Fit Index (CFI), Tucker–Lewis Index (TLI), and Root Mean Square Error of Approximation (RMSEA).

**Internal Consistency:** Assessed using Cronbach’s alpha, with values ≥0.70 considered acceptable. Omega coefficient was also calculated as a complementary measure, with values ≥0.70 indicating adequate consistency.

**Split-Half Reliability:** Assessed by correlating scores from odd- and even-numbered items. The correlation was adjusted using the Spearman–Brown formula to estimate the reliability of the full instrument. A threshold of ≥0.70 was used to define acceptable reliability.

**Criterion Validity:** A single global item (overall satisfaction with hospital food) was used as a closely related anchor to examine the expected association with the PREM total score, given the absence of a gold standard for patient experience with food services. We prespecified the following hypotheses: (i) a strong positive association between the PREM total score and the global satisfaction item (expected Spearman’s rho ≥ 0.60); (ii) a monotonic increase in PREM scores across ordered categories of the global item; and (iii) good discrimination of high global satisfaction (score 4–5 on a 5-point scale) by the PREM (expected AUC ≥ 0.75). We computed Spearman’s rho with 95% confidence intervals via bias-corrected bootstrapping (10,000 resamples), tested for monotonic trend using the Jonckheere–Terpstra test, and estimated the ROC curve with AUC and the Youden-optimal cut-off. In sensitivity analyses, we fitted an ordinal logistic regression of the global satisfaction item on the PREM total score adjusting for diet type and hospital unit, and computed partial Spearman correlations adjusting for the same covariates.

**Cross-Cultural Validity:** Ensured through adaptation of items to reflect the diversity of the patient population typically served at the HACLE. Items were developed and reviewed by the hospital’s psychology team to ensure sensitivity to sociocultural differences.

**Responsiveness:** Evaluated by comparing PREM scores across patients receiving different types of diets.

**Interpretability:** To facilitate interpretation of total scores, respondents were categorized into three groups based on the empirical distribution. Percentiles P33 and P66 of the total score (range: 0–32) were calculated as 17.3 and 21.3, respectively. Based on these cut-offs, three categories were defined: low score (0–17.3), medium score (17.4–21.3), and high score (>21.3).

**Sensitivity Analysis:** We compared PREM scores across respondent types (patients, relatives, paid caregivers) to assess whether proxy completion influenced results. Similarly, we compared mean PREM scores between the 4-point period (2011–2023) and the 5-point period (2024–2025) to verify that the scale harmonization did not alter the interpretation of trends or dimension-level differences.

**Missing Data Analysis:** We examined the presence of missing data across different years, hospital units, and diet types to evaluate whether missingness introduced potential bias.

### 2.8. Study 2: Trend and Pattern Analysis

Temporal trends in PREM scores between 2011 and 2025 were explored using descriptive statistics (frequencies, means, and standard deviations) and inferential models. To assess item-level changes over time, simple linear regressions were applied, along with segmented linear regression models (interrupted time series) to detect the impact of two institutional milestones: the change in catering provider in 2022 and the introduction of new food distribution carts in 2025. The Mann–Whitney U test was used to compare total PREM scores between hospital units (Comprehensive Medical Area vs. Mental Health), and the Kruskal–Wallis test was employed to examine satisfaction across different diet types. Post hoc pairwise comparisons were conducted using Dunn’s test with Bonferroni correction. A significance level of *p* < 0.05 was adopted for all analyses. Statistical analyses were conducted using R software (R 4.5.1, Foundation for Statistical Computing, Vienna, Austria, 2025).

### 2.9. Use of Generative Artificial Intelligence

ChatGPT (OpenAI, San Francisco, CA, USA) was used to assist in the English translation of the manuscript text. The authors reviewed and edited the content to ensure accuracy and clarity.

## 3. Results

The analysis of the time-trend analysis using PREM was based on a total of 1618 surveys collected between 2011 and 2025. Among them, 91 (5.6%) contained at least one unanswered item, and 60 (3.7%) had multiple missing responses. Regarding diet type based on texture or format, the majority of patients received a regular diet (60.4%, *n* = 978), followed by pureed diet (21.8%, *n* = 353) and soft diets (16.4%, *n* = 265). Less frequent diets included liquid diets (0.1%, *n* = 1) and soft diet excluding foods that pose a choking hazard (1.2%, *n* = 20). Additionally, diets were classified according to their therapeutic purpose. A normal diet was prescribed to 33.7% of patients (*n* = 546), a hypocaloric diet to 31.8% (*n* = 514), and a fortified diet to 30.6% (*n* = 495). Less frequent therapeutic diets included hepatic diets (0.6%, *n* = 10), renal diets (0.8%, *n* = 13), and soft diets with digestive protection (1.4%, *n* = 22). Regarding the distribution of total PREM scores, 548 patients (33.9%) were classified as having a low score (0–17.3 points), 476 (29.4%) had a medium score (17.4–21.3 points), and 514 (31.8%) had a high score (>21.3 points). A total of 80 participants (4.9%) were excluded from the final score analysis due to incomplete responses on the eight items required to calculate the total score.

### 3.1. Study 1. Psychometric Properties of the PREM

#### 3.1.1. Item Analysis

The item-total correlation analysis showed that all items exceeded the acceptable threshold of 0.30, indicating adequate contribution to the internal consistency of the questionnaire. In all cases, internal consistency improved in the 8-item version compared to the original 9-item version (Table 1).

#### 3.1.2. Internal Consistency

The analysis revealed high overall internal consistency, supported by a Cronbach’s alpha of 0.85. The Omega coefficient (0.87) further confirmed the reliability of the scale.

When analyzing consistency at the factor level, Factor 1 showed a satisfactory Omega value of 0.87, whereas Factor 2 presented an Omega of 0.50.

#### 3.1.3. Reliability

Internal reliability assessed using the split-half method showed a correlation between halves of r = 0.74, and a Spearman–Brown coefficient of 0.85, indicating good internal consistency of the instrument (Table 2).

#### 3.1.4. Construct Validity

##### Exploratory Factor Analysis (EFA)

**Figure 1:**
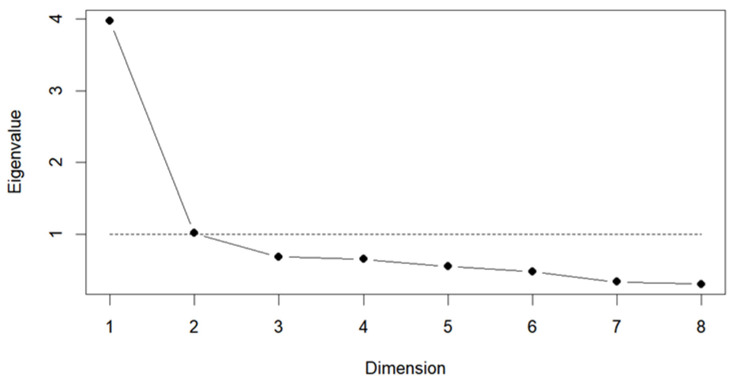
Determination of the optimal number of factors. Dots represent the eigenvalues for each dimension, and the line connects these values to illustrate the trend across components. The dashed horizontal line indicates the eigenvalue threshold of 1.

The optimal number of factors to extract was determined to be two (KMO = 0.922; Bartlett’s test of sphericity χ^2^ = 5244.953, *df* = 45, *p* < 0.0001), supporting the suitability of the dataset for factor analysis (Figure 1).

The resulting factor structure from the exploratory factor analysis (EFA) included the items Quality (0.87), Presentation (0.55), Cooking (0.73), Taste (0.90), Variety (0.68), and Temperature (0.37) loading on the first factor. The second factor included Timing (0.59) and Cleanliness (0.61) (Table 3).

**Figure 2:**
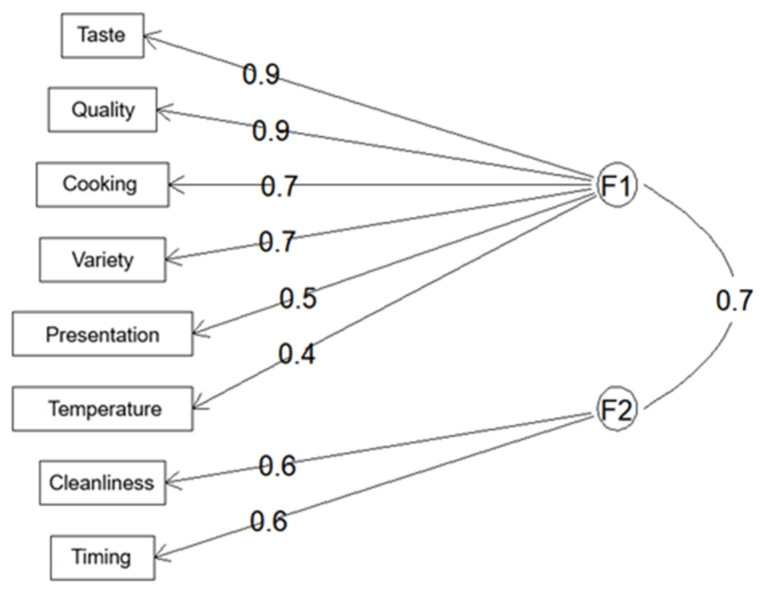
Exploratory factor analysis model showing two latent factors (F1: food-related attributes, F2: service-related attributes).

The first factor was associated with food-related attributes, including quality, presentation, preparation, taste, variety, and serving temperature. In contrast, the second factor encompassed aspects related to service, such as timing of meal distribution and cleanliness. These two factors were moderately correlated (r = 0.70) (Figure 2).

##### Confirmatory Factor Analysis (CFA)

**Figure 3:**
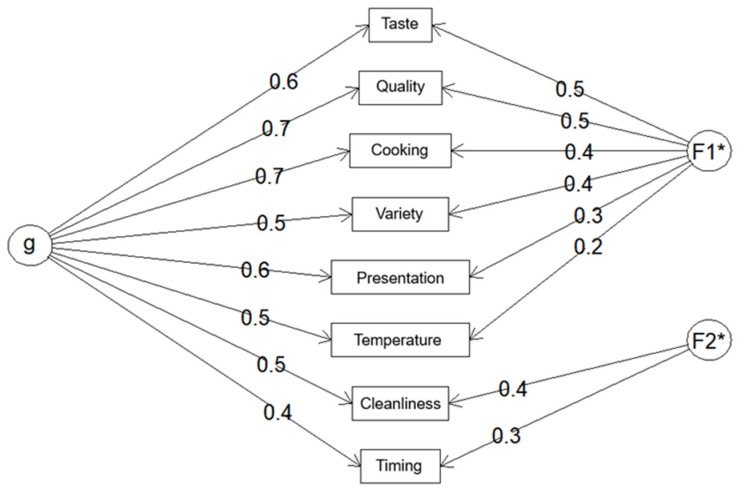
Confirmatory factor analysis model showing item loadings on the general factor (g) and specific latent factors F1 (food-related aspects) and F2 (service-related aspects). Asterisk (*) indicates parameters fixed for model identification.

In the confirmatory factor analysis model (Figure 3), standardized factor loadings were estimated for each item on the general factor (“g”) and on two specific latent factors (F1: food-related aspects; F2: service-related aspects). While most items showed adequate associations with their corresponding factors, a few items displayed lower loadings, indicating weaker contributions to the latent constructs. Specifically, the items Temperature, Presentation, and Timing showed relatively low factor loadings (ranging from 0.20 to 0.37) and reduced communalities. In addition, Temperature demonstrated high complexity (1.84), suggesting a less clear factorial structure. Despite these findings, the aforementioned items were retained in the final version of the instrument due to their clinical relevance, contribution to overall service perception, and their acceptable correlation with the total questionnaire score.

The fit indices indicated that the two-factor model achieved acceptable convergence and structural robustness. Overall, the model demonstrated a satisfactory fit, meeting conventional acceptability thresholds for goodness-of-fit indices (Table 4).

#### 3.1.5. Sensitivity Analysis

We compared PREM scores across respondent types (patients, relatives, paid caregivers) to assess whether proxy completion influenced results. Although patients generally reported lower scores, no major changes were observed, suggesting that proxy completion had minimal influence on the overall PREM score (Supplementary Material Table S2). Similarly, we compared mean PREM scores between the 4-point period (2011–2023) and the 5-point period (2024–2025) to verify that the scale harmonization did not alter the interpretation of trends or dimension-level differences. The results showed that the scale harmonization did not lead to major changes in the score trends or dimension-level differences, and the conclusions of the study remained unchanged (Supplementary Materials Table S3).

#### 3.1.6. Missing Data Analysis

We examined the presence of missing data across different years, hospital units, and diet types to evaluate whether missingness introduced potential bias. The results showed that the missing data were relatively low and did not follow a systematic pattern across years, hospital units, or diet types, (Supplementary Materials Tables S4–S6).

### 3.2. Study 2: Temporal Trends

**Figure 4:**
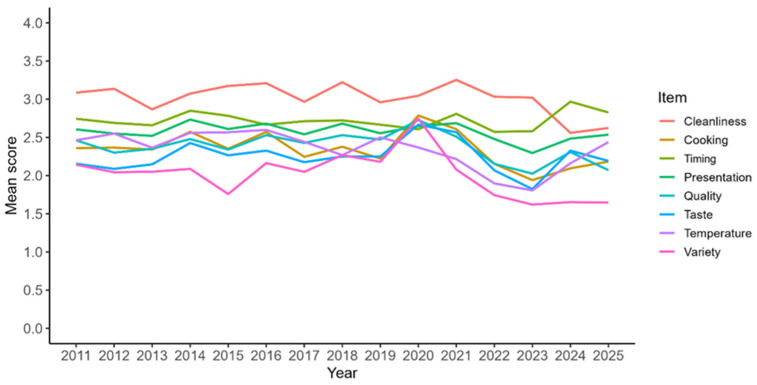
Trends in average item scores from 2011 to 2025.

Regarding the temporal evolution of the scores obtained for each evaluated item, a general stability was observed over the years, although with a slight downward trend in certain categories—particularly Variety, Temperature, and Taste (Figure 4). In 2023, a pronounced and widespread decline occurred across all items, followed by a visible recovery in 2024 and 2025, except for Variety, which remained consistently low. The item Cleanliness recorded the highest average scores over the study period, with peaks in 2021, 2018, and 2016, although it was surpassed by Timing from 2024 onwards. In contrast, Variety consistently received the lowest ratings, especially during the last three years.

**MILESTONE 1:** Change in Catering Company

The change in catering provider (reflected in the data from 2022 onwards) coincided with a significant decline in perceived quality across several aspects of the hospital food service, particularly in food-related attributes such as quality, preparation, presentation, temperature, variety, and taste. Only the item Timing remained stable. Moreover, prior to the change, some dimensions—such as Taste— had shown an upward trend prior to 2022, which shifted to a downward pattern after this point (Table 5).

**MILESTONE 2:** Change in Meal Distribution Carts

Prior to 2025, the score for the Temperature item exhibited a significant negative trend (β = −0.0375, *p* < 0.0001), indicating a progressive decline in patient perception of this aspect over time. Following the introduction of new food distribution carts in 2025—designed to better retain food temperature—a significant positive trend was observed (β = 0.3909, *p* = 0.0032), suggesting a temporal association between the implementation of the new carts and higher temperature ratings (Table 6).

The temporal trend analysis revealed a significant decline in the scores of several questionnaire items, including Quality, Cooking, Variety, Temperature, Cleanliness, and the overall score, reflecting a progressive downward pattern in patient perceptions of these aspects of hospital food service between 2011 and 2025. In contrast, the items Taste, Presentation, and Timing did not show significant changes, suggesting a stable patient evaluation of these service components over the analyzed period (Table 7).

#### 3.2.1. Comparison of Mean Scores by Hospital Units

The overall mean satisfaction score was slightly higher in the Comprehensive Medical Area (19.65 ± 5.66) compared to the Mental Health Unit (19.50 ± 5.83). However, this difference was not statistically significant (*p* = 0.4310), suggesting no relevant differences in overall satisfaction between the two hospital units (Table 8).

#### 3.2.2. Criterion Validity

The PREM total score correlated strongly and positively with the global satisfaction item (Spearman’s rho = 0.80; 95% CI: 0.77–0.82; *p* < 0.001), in line with a prior hypothesis. PREM scores increased monotonically across ordered categories of the global item (Jonckheere–Terpstra JT = 509,620, *p* < 0.001; median PREM scores rose consistently across categories [6,10,11,12]). The PREM showed good discriminatory ability for high global satisfaction (AUC = 0.94; 95% CI 0.92–0.95; the Youden-optimal cut-off of 14.5, yielded sensitivity = 88.1% and specificity = 86.1%). The association remained robust after adjustment for diet type and unit in ordinal logistic regression (adjusted odds ratio per 1-point increase in PREM = 1.82; 95% CI 1.74–1.91; *p* < 0.001) and in partial correlations (partial rho = 0.70, *p* < 0.001).

#### 3.2.3. Comparison of Mean Scores by Type of Diet

Patients receiving a pureed diet reported the highest average satisfaction score (21.18 ± 5.28), followed by those on a soft diet (19.61 ± 5.43) and a regular diet (19.08 ± 5.86). In contrast, the group on a soft diet excluding foods that pose a choking hazard had the lowest mean score (17.25 ± 5.49). The Kruskal–Wallis test showed that these differences were statistically significant (χ^2^ = 34.53; *p* < 0.0001), indicating that the type of diet administered had a meaningful impact on overall satisfaction with hospital food services (Table 9).

Post hoc pairwise comparisons using Dunn’s test revealed that the pureed diet was rated significantly higher than the regular diet (*p* < 0.0001) and the soft diet (*p* = 0.0073). The comparison between the pureed diet and the soft diet without food textures that pose a risk showed a marginally significant difference (*p* = 0.088). No significant differences were observed among the other diet combinations.

## 4. Discussion

This study provides a 15-year time-trend perspective on patient experience with hospital food in a long-term and chronic care hospital (HACLE) in Spain. It also presents a patient-reported experience measure (PREM) specifically developed to assess food-related patient experience in HACLEs, with potential applicability to other long-term care and social-healthcare settings. In this context, it is important to note that hospital diets in the study center are designed in accordance with ESPEN guideline recommendations. Nutritional requirements are individually assessed, and dietary plans are tailored to provide the energy and nutrient intake required by each patient. The regular diet, which is the most commonly prescribed, is designed to provide approximately 30 kcal/kg body weight/day and 1.0–1.2 g protein/kg body weight/day, together with adequate amounts of macronutrients, vitamins, and trace elements. Puréed diets and soft diets excluding foods that may pose a choking risk are used as texture-modified dietary options, while therapeutic diets are prescribed according to patients’ clinical conditions and specific nutritional needs [13].

First, the results suggest that the factorial structure of the questionnaire supports the existence of two main dimensions—food quality and service conditions—with adequate internal consistency and structural validity, thus endorsing its use as a monitoring tool to help detect malnutrition in this population [9,14]. This structure aligns with previous findings reported in other hospital-based studies. While the instrument demonstrated strong overall psychometric performance, the total score showed greater robustness than the subscale structure, particularly given the somewhat lower reliability of Factor 2. Even so, the scale proved sensitive to real-world organizational changes, reinforcing its relevance for clinical and managerial practice. The instrument developed and validated in this study not only demonstrated strong psychometric properties but also proved sensitive to real-world organizational changes, reinforcing its relevance for clinical and managerial practice. Taken together, these findings highlight that food provision in long-term care settings is not only a clinical matter but also shapes patients’ overall perception of the care environment [4].

Second, the findings from the 15-year repeated cross-sectional analysis reveal heterogeneous perceptions of hospital food, in which factors such as the prescribed diet type and the organization of food services play a significant role in shaping patient experience. Pureed diets received higher satisfaction ratings, suggesting that perceived nutritional adequacy contributes to a sense of personalized care. This may also be explained by the fact that patients perceive pureed textures as facilitating food intake and find them to be appropriately prepared from an organoleptic standpoint. In contrast, structural changes in service provision—such as the replacement of the catering company—had a negative impact on the evaluation of several food-related attributes, highlighting the sensitivity of the patient experience to organizational decisions.

Unlike most previous studies, which have focused on acute care hospitals [10], this work is based on an instrument specifically adapted to the Spanish context and validated for medium- and long-term care hospitals, which often lack tools and methodologies to assess patient experience in these settings. In this case, the focus is on a particularly relevant issue for clinical outcomes: maintaining adequate nutritional status [15].

Our findings are consistent with previous research showing that patient satisfaction with hospital food services is significantly influenced by the type of diet and the sensory attributes of the meals, such as appearance and variety, which have also been linked to increased plate waste and potential nutritional risk [16]. As highlighted in studies conducted in acute care hospitals, both the temperature and texture of meals are important attributes valued by patients [17]. These results align with previous studies on texture-modified diets, where appearance and variety were identified as key predictors of patient satisfaction and plate waste, underscoring the importance of sensory quality in preventing malnutrition and improving care outcomes [10,16,18].

The type of diet, the patient’s appetite, and other contextual factors are known to influence how patients evaluate the food and food service they receive during hospitalization [11]. One of the most consistent findings was the persistently low rating of menu variety, which showed no improvement over time. This aligns with previous studies indicating that longer hospital stays are negatively associated with satisfaction with hospital meals [19,20]. Both factors play a particularly important role in long-term and chronic care hospitals (HACLEs), supporting the notion that attention to the patient’s experience with food is a key determinant of their well-being and nutritional status [21].

The temporal analysis revealed the sensitivity of patients’ perceptions to specific organizational changes. The change in catering company in 2022 was associated with a significant decline in multiple dimensions of the food experience, whereas the introduction of new food distribution carts in 2025 led to a notable improvement in how patients rated food temperature. These organizational changes were not accompanied by other significant modifications, such as changes in menus, staff composition, or service schedules. This pattern suggests that seemingly logistical interventions can have a direct impact on the patient experience, and that such impact can be effectively measured with appropriate tools.

Previous studies adopting multidisciplinary approaches have shown that targeted improvements in hospital food services can lead to better nutritional status, increased food intake, enhanced patient satisfaction, and improved quality of life [22]. The findings of our study reinforce this perspective, highlighting how specific organizational factors—such as menu planning, distribution systems, and responsiveness to individual dietary needs—have a direct impact on patients’ experience. Based on these results, several practical implications emerge, consistent with recommendations from previous research [23]. enhancing the sensory quality of meals (taste, smell, variety), ensuring timely and temperature-appropriate distribution, training staff in communication and patient-centered care, and systematically incorporating patient feedback into quality improvement cycles. Moreover, addressing individual preferences and dietary restrictions not only contributes to nutritional adequacy but also strengthens patients’ perception of being respected and cared for. The validated PREM scale presented in this study offers a reliable tool to monitor these dimensions and guide targeted interventions across similar long-term care or institutional settings.

### 4.1. Practical Implications

One of the main contributions of this study is the validation of a culturally adapted PREM questionnaire specifically focused on the food experience in long-term care settings, developed and tested in a real-world context. This scale can be employed by other hospitals with similar characteristics and, with minimal adaptations, could also be applied in residential care facilities, intermediate care units, mental health centers, or other institutional settings where food provision is an integral part of care and person-centered approaches [12].

The fact that the instrument clearly detected the effects of organizational changes in menu planning and distribution underscores its value as a practical tool for quality evaluation and evidence-based decision-making. It also enables the identification of dietary profiles associated with higher or lower satisfaction levels, thus informing the personalization of nutritional interventions.

In response to the PREM results obtained, several improvements were introduced from 2025 onward. These included the design of new seasonal menus, with special attention to preserving optimal food temperature during transport from the kitchen to patients’ rooms. Additionally, efforts were made to diversify soft diets by eliminating high-risk items while maintaining variety and palatability. These changes reflect a direct application of patient feedback to redesign meal planning and service logistics, reinforcing the PREM tool’s utility for guiding actionable improvements in care delivery.

The validated PREM scale developed in this study offers a practical tool for systematically monitoring patient experience with hospital food services. Its integration into quality improvement cycles—particularly through structured PDCA (Plan-Do-Check-Act) models—can facilitate continuous evaluation and timely adjustments. We recommend the quarterly review of PREM results by a multidisciplinary clinical-nutritional committee, allowing the identification of emerging issues and the prioritization of corrective actions. In particular, targeted nutritional audits should be conducted for diets consistently associated with lower satisfaction scores, such as soft diets without dual textures. These audits should not only assess nutritional adequacy but also include a sensory evaluation of meals (e.g., taste, appearance, variety), to ensure that therapeutic constraints do not compromise the perceived quality of care. This approach enables responsive, patient-centered dietary planning and supports institutional learning over time. Such evaluations should be carried out in parallel with routine nutritional assessment and ongoing institutional strategies to prevent and correct malnutrition, as recent evidence in older hospitalized patients highlights that malnutrition is associated with poorer function and quality of life and may remain underdetected during hospitalization if not actively assessed [24].

### 4.2. Strengths

This study provides a rare 15-year repeated cross-sectional perspective, covering a 15-year period and allowing for an in-depth analysis of changes in patient experience related to hospital food services in a medium- and long-term care facility. Secondly, it validates a culturally adapted, context-specific PREM instrument with sound psychometric properties, which has proven sensitive to organizational changes. This capacity to detect significant variations in patient perceptions makes it a valuable tool for both ongoing assessment and quality improvement. Finally, the study offers empirical evidence reinforcing the importance of attending not only to the sensory attributes of food but also to the organizational aspects of food service—elements that are often underestimated in long-term care hospitals.

### 4.3. Limitations

As a retrospective observational study, this design does not allow for definitive causal inferences between organizational changes and variations in patient perceptions. Although segmented time series models were used to enhance the robustness of the analysis, unmeasured confounding variables may still have influenced the results. The associations observed around the change in catering company and the introduction of new meal-distribution carts should be interpreted as exploratory and hypothesis-generating. Given the annual cross-sectional design, the single-center setting, and the very limited post-intervention window for the 2025 cart implementation, no causal inferences can be drawn from these temporal patterns. Additionally, the data were collected from a single facility, which may limit the generalizability of the findings to other settings or regions. Patient participation was contingent on their clinical and cognitive status at the time of data collection, potentially introducing selection bias. It is also possible that some patients may have responded more than once due to multiple admissions; however, in a long-term care hospital, repeated admissions are not very common. While the proxy used is not a gold standard, its content proximity and the robustness of results across model adjustments and scale versions increase confidence in the PREM’s criterial performance. The global satisfaction item used as an anchor was conceptually close to the PREM content. Therefore, the observed association should be interpreted as supportive only. Although missing data were relatively low and did not show systematic patterns across years, hospital units, or diet types, the analyses relied on complete case data. This approach may still introduce some degree of bias, and the results should therefore be interpreted with caution. Future work could examine predictive relationships with external outcomes (e.g., plate waste, nutritional risk, or length of stay). Finally, although the questionnaire demonstrated good content and structural validity, further research should explore its convergent validity and its applicability in other long-term or social care settings beyond the hospital studied.

## 5. Conclusions

The experience of hospitalized patients with food services in long-term care is a key component of person-centered care and a relevant indicator of care quality. This study demonstrates that such experience can be reliably measured using a validated PREM instrument and is significantly influenced by organizational factors such as diet type and distribution systems. The tool developed enables the identification of areas for improvement and supports decision-making aimed at enhancing patient well-being and nutritional status. Its implementation in similar facilities could contribute to more personalized, responsive care that is sensitive to the dietary needs and preferences of hospitalized individuals. Furthermore, future studies could explore how malnutrition and patient experience are interrelated, providing valuable insights into how these factors influence each other.

## Figures and Tables

**Table 1:** Item-total correlations for the 9-item and 8-item versions of the PREM.

9-Item Version	Item-Total Correlation	8-Item Version	Item-Total Correlation
Food quality	0.74	Food quality	0.78
Presentation of meals	0.63	Presentation of meals	0.68
Cooking of the food	0.72	Cooking of the food	0.72
Satisfaction with the taste	0.71	Satisfaction with the taste	0.76
Variety of the diet	0.58	Variety of the diet	0.66
Food temperature	0.52	Food temperature	0.60
Portion size	0.16	Portion size	–
Timing of meal distribution	0.36	Timing of meal distribution	0.42
Dish and cutlery cleanliness	0.43	Dish and cutlery cleanliness	0.49

**Table 2:** Reliability Analysis of the Items.

Item	General	Factor 1	Factor 2	Communality	Unexplained Variance	Unique Reliability
Food quality	0.67	0.50	--	0.70	0.30	0.64
Presentation of meals	0.59	0.32	--	0.47	0.53	0.76
Cooking	0.68	0.42	--	0.64	0.36	0.72
Satisfaction with the taste	0.63	0.52	--	0.68	0.32	0.60
Variety of the diet	0.51	0.39	--	0.42	0.58	0.62
Food temperature	0.52	0.21	--	0.34	0.66	0.79
Timing of meal distribution	0.43	--	0.34	0.30	0.70	0.61
Dish and cutlery cleanliness	0.50	--	0.35	0.38	0.62	0.67

**Table 3:** Exploratory Factor Analysis with Two Factors (F1, F2).

Item	Factor 1	Factor 2	Communality	Unexplained Variance	Complexity
Food quality	0.87	−0.05	0.70	0.30	1.01
Presentation of meals	0.54	0.18	0.47	0.53	1.21
Cooking	0.73	0.11	0.64	0.36	1.04
Satisfaction with the taste	0.90	−0.12	0.68	0.32	1.03
Variety of the diet	0.68	−0.06	0.42	0.58	1.01
Food temperature	0.37	0.27	0.34	0.66	1.84
Timing of meal distribution	−0.06	0.59	0.30	0.70	1.02
Dish and cutlery cleanliness	0.00	0.61	0.38	0.62	1.00

**Table 4:** Model Fit Indices.

Index	Value	Threshold
Comparative Fit Index (CFI)	0.981	≥0.95 (excellent), ≥0.90 (acceptable)
Tucker–Lewis Index (TLI)	0.972	≥0.95 (excellent), ≥0.90 (acceptable)
Root Mean Square Error of Approximation (RMSEA)	0.055	<0.06 (excellent), ≤0.08 (acceptable)
Standardized Root Mean Square Residual (SRMR)	0.029	<0.05 (excellent), ≤0.08 (acceptable)

**Table 5:** Segmented Time Series Analysis: Impact of the Catering Company Change on Satisfaction with the Food Service (2011–2025).

Item	Before 2022 β	Before 2022 *p*-Value	After 2022 β	After 2022 *p*-Value
Food quality	0.0196	0.0361	−0.4681	<0.0001
Presentation of meals	0.0084	0.3197	−0.2229	0.0165
Cooking	0.0149	0.1210	−0.4396	<0.0001
Satisfaction with the taste	0.0370	0.0003	−0.4556	0.0001
Variety of the diet	0.0223	0.0612	−0.6481	<0.0001
Food temperature	−0.0078	0.4324	−0.2886	0.0087
Timing of meal distribution	0.0019	0.7990	0.0233	0.7790
Dish and cutlery cleanliness	0.0009	0.9031	−0.3012	0.0003

**Table 6:** Segmented Time Series Analysis: Impact of the Change in Meal Distribution Carts on Satisfaction with Food Temperature (2011–2025).

Item	β (Before 2025)	*p*-Value (Before 2025)	β (After 2025)	*p*-Value (After 2025)
Temperature	−0.0375	<0.0001	0.3909	0.0032

**Table 7:** Temporal Trend Analysis by Item: Slopes, Statistical Significance, and Direction of Change (2011–2025).

Item	β	*p*-Value	Trend
Food quality	−0.013	0.0215	Significant decrease
Presentation of meals	0.005	0.4587	No significant change
Cooking	−0.007	0.1544	No significant change
Satisfaction with the taste	−0.016	0.0068	Significant decrease
Variety of the diet	−0.024	0.0016	Significant decrease
Food temperature	−0.028	<0.0001	Significant decrease
Timing of meal distribution	0.004	0.4450	No significant change
Dish and cutlery cleanliness	−0.020	<0.0001	Significant decrease
Total score	−0.096	0.0025	Significant decrease

**Table 8:** Mean Satisfaction Score by Hospital Unit.

Unit	Mean Total Score (M ± SD)	
Comprehensive Medical Area (CMA)	19.65 ± 5.66	
Mental Health (MH)	19.50 ± 5.83	
Group Comparison (Mann–Whitney U test)	W	*p*-value
Hospital Unit	296,409	0.4310

**Table 9:** Mean Overall Satisfaction by Type of Diet Administered.

Type of Diet		Mean Overall Score (M ± SD)	
Regular Diet		19.08 ± 5.86	
Soft Diet		19.61 ± 5.43	
Pureed diet		21.18 ± 5.28	
Soft diet excluding foods that pose a choking hazard		17.25 ± 5.49	
Group Comparison (Kruskal–Wallis Test)	Degrees of freedom (*df*)	χ^2^	*p*-value
	4	34.53	<0.0001

## Data Availability

The data presented in this study are available on request from the corresponding author due to institutional confidentiality policies and data protection regulations.

## Supplementary Materials

The following supporting information can be downloaded at: https://www.mdpi.com/article/10.3390/nu18081246/s1, Questionnaire S1: Original 9-item Nutrition PREM (2011–2023 version). Questionnaire S2: Updated 8-item Nutrition PREM (2024–2025 version). Table S1. Distribution of Respondent Types. Table S2. Sensitivity Analysis: Mean PREM Scores by Respondent Type. Table S3. Sensitivity Analysis: Mean PREM scores Before and After Scale Change. Table S4. Missing Data Analysis by Year. Table S5. Missing Data Analysis by Type of Diet. Table S6. Missing Data Analysis by Hospital Unit.

## Abbreviations

The following abbreviations are used in this manuscript:

AUC	Area Under the Curve
CA	California
CFA	Confirmatory Factor Analysis
CFI	Comparative Fit Index
CI	Confidence Interval
CMA	Comprehensive Medical Area
COSMIN	Consensus-based Standards for the Selection of Health Measurement Instruments
*df*	Degrees of Freedom
EFA	Exploratory Factor Analysis
HACLE	Long-Term and Chronic Care Hospital
JT	Jonckheere–Terpstra
KMO	Kaiser–Meyer–Olkin
MH	Mental Health
MeSH	Medical Subject Headings
PREM	Patient-Reported Experience Measure
RMSEA	Root Mean Square Error of Approximation
ROC	Receiver Operating Characteristic
SD	Standard Deviation
SRMR	Standardized Root Mean Square Residual
TLI	Tucker–Lewis Index
α	Cronbach’s Alpha
ω	Omega Coefficient
