# Supplementary material for: Fifteen Years of Patient Experience with Hospital Food in a Spanish Long-Term Care Hospital
Source: Nutrients. 2026 Apr 15;18(8):1246. doi: 10.3390/nu18081246 (PMC13119420; doi:10.3390/nu18081246)
Supplement: Supplementary file 1 [file nutrients-18-01246-s001.zip › nutrients-4225977-supplementary.pdf]

Hospital Pare Jofré

**Questionnaire S1.** Original 9-item Nutrition PREM (2011–2023 version)

OPINION QUESTIONNAIRE. FOOD AND CATERING SERVICE

This questionnaire is aimed at improving the type of diet each patient receives. Your response will be treated ano-ymously and will not affect the quality of the hospital care you receive. We kindly ask you to answer sincerely. Thank you very much.

| | |
|---|---|
| 1. WHAT DO YOU THINK ABOUT THE QUALITY OF THE FOOD OFFERED TO YOU? | ☐ Very good<br>☐ Good<br>☐ Fair<br>☐ Poor |
| 2. WHAT DO YOU THINK ABOUT THE PRESENTATION OF THE DISHES? | ☐ Very good<br>☐ Good<br>☐ Fair<br>☐ Poor |
| 3. WHAT DO YOU THINK ABOUT HOW THE FOOD IS COOKED? | ☐ Very good<br>☐ Good<br>☐ Fair<br>☐ Poor |
| 4. DO YOU LIKE THE FOOD YOU ARE SERVED? | ☐ A lot<br>☐ Quite a bit<br>☐ Little<br>☐ Very little |
| 5. HOW DO YOU FIND THE VARIETY OF THE DIET? | ☐ Very varied<br>☐ Sufficient<br>☐ Insufficient<br>☐ Not very varied |
| 6. HOW IS THE TEMPERATURE OF THE FOOD WHEN IT REACHES YOU? | ☐ Perfect<br>☐ Good<br>☐ Fair<br>☐ Poor |

Continue →

| | |
|---|---|
| 7. HOW DO YOU CONSIDER THE QUANTITY OF THE FOOD? | ☐ Too much<br>☐ Adequate<br>☐ Scarce<br>☐ Very scarce |
| 8. DO YOU THINK THE MEAL DELIVERY SCHEDULE IS CONSISTENT? | ☐ Always<br>☐ Yes<br>☐ Sometimes<br>☐ Never |
| 9. HOW DO YOU FIND THE CLEANLINESS OF THE DISHES AND CUTLERY? | ☐ Very good<br>☐ Good<br>☐ Fair<br>☐ Poor |
| 10. OVERALL OPINION OF THE FOOD SERVICE | ☐ Very good<br>☐ Good<br>☐ Fair<br>☐ Poor |
| Suggestions: | |

Overall, how satisfied were you?

| Not at all satisfied | 0 | 1 | 2 | 3 | 4 | 5 | 6 | 7 | 8 | 9 | 10 | Completely satisfied |
|---|---|---|---|---|---|---|---|---|---|---|---|---|
| | | | | | | | | | | | | |

PERSON COMPLETING THE SURVEY:

☐ PATIENT

☐ PAID CAREGIVER

☐ FAMILY MEMBER

TYPE OF DIET:

☐ WITH SALT

☐ SALT-FREE

☐ REGULAR

☐ SOFT

☐ HIGH-CALORIE

☐ LOW-CALORIE

☐ PURÉED

☐ LIQUID

☐ HEPATIC

☐ RENAL

Hospital Pare Jofré

**Questionnaire S2.** Updated 8-item Nutrition PREM (2024–2025 version)

OPINION QUESTIONNAIRE. FOOD AND CATERING SERVICE

This questionnaire is aimed at improving the type of diet each patient receives. Your response will be treated anonymously and will not affect the quality of the hospital care you receive. We kindly ask you to answer sincerely. Thank you very much.

| | |
|---|---|
| 1. WHAT DO YOU THINK ABOUT THE QUALITY OF THE FOOD OFFERED TO YOU? | ☐ Very good<br>☐ Good<br>☐ Adequate<br>☐ Fair<br>☐ Poor |
| 2. WHAT DO YOU THINK ABOUT THE PRESENTATION OF THE DISHES? | ☐ Very good<br>☐ Good<br>☐ Adequate<br>☐ Fair<br>☐ Poor |
| 3. WHAT DO YOU THINK ABOUT HOW THE FOOD IS COOKED? | ☐ Very good<br>☐ Good<br>☐ Adequate<br>☐ Fair<br>☐ Poor |
| 4. DO YOU LIKE THE FOOD YOU ARE SERVED? | ☐ A lot<br>☐ Quite a bit<br>☐ Somewhat<br>☐ Little<br>☐ Very little |
| 5. HOW DO YOU FIND THE VARIETY OF THE DIET? | ☐ Very varied<br>☐ Quite varied<br>☐ Adequate<br>☐ Not very varied<br>☐ Insufficiently varied |

Continue →

| | |
|---|---|
| 6. HOW IS THE TEMPERATURE OF THE FOOD WHEN IT REACHES YOU? | ☐ Perfect<br>☐ Quite good<br>☐ Adequate<br>☐ Fair<br>☐ Poor |
| 7. DO YOU THINK THE MEAL DELIVERY SCHEDULE IS CONSISTENT? | ☐ Always<br>☐ Often<br>☐ Sometimes<br>☐ Occasionally<br>☐ Never |
| 8. HOW DO YOU FIND THE CLEANLINESS OF THE DISHES AND CUTLERY? | ☐ Very good<br>☐ Good<br>☐ Adequate<br>☐ Fair<br>☐ Poor |
| 9. OVERALL OPINION OF THE FOOD SERVICE | ☐ Very good<br>☐ Good<br>☐ Adequate<br>☐ Fair<br>☐ Poor |

Suggestions:

Overall, how satisfied were you?

| Not at all satisfied | 0 | 1 | 2 | 3 | 4 | 5 | 6 | 7 | 8 | 9 | 10 | Completely satisfied |
|---|---|---|---|---|---|---|---|---|---|---|---|---|
| | | | | | | | | | | | | |

PERSON COMPLETING THE SURVEY:

☐ PATIENT

☐ PAID CAREGIVER

☐ FAMILY MEMBER

TYPE OF DIET:

☐ WITH SALT

☐ SALT-FREE

☐ REGULAR

☐ SOFT

☐ HIGH-CALORIE

☐ LOW-CALORIE

☐ PURÉED

☐ LIQUID

☐ HEPATIC

☐ RENAL

Hospital Pare Jofré

**Table S1**. Distribution of Respondent Types

| Respondent | N | % |
|---|---|---|
| **Patient** | 1294 | 80.0% |
| **Paid caregiver** | 56 | 3.5% |
| **Relative** | 268 | 16.6% |

**Table S2**. Sensitivity Analysis: Mean PREM Scores by Respondent Type

| | | | | Paid Caregivers vs Patients | | Relatives vs Patients | |
|---|---|---|---|---|---|---|---|
| Dimension | Patients | Paid caregivers | Relatives | Absolute diff. | Relative diff. | Absolute diff | Relative diff. |
| Food quality | 1.7939 | 2.0909 | 2.0489 | 0.2970 | 16.6% | 0.2550 | 14.2% |
| Presentation of meals | 1.9611 | 2.0179 | 2.1798 | 0.0568 | 2.9% | 0.2187 | 11.2% |
| Cooking of the food | 1.7601 | 2.0179 | 2.0792 | 0.2577 | 14.6% | 0.3191 | 18.1% |
| Satisfaction with the taste | 1.7143 | 1.7857 | 1.8517 | 0.0714 | 4.2% | 0.1374 | 8.0% |
| Variety of the diet | 1.5352 | 1.5893 | 1.7015 | 0.0568 | 3.7% | 0.1690 | 11.0% |
| Food temperature | 1.7697 | 2.1250 | 2.1343 | 0.3553 | 20.1% | 0.3646 | 20.6% |
| Portion size | 2.1188 | 2.1250 | 2.2276 | 0.0062 | 0.3% | 0.1088 | 5.1% |
| Timing of meal distribution | 2.3259 | 2.3571 | 2.4403 | 0.0312 | 1.3% | 0.1144 | 4.9% |
| PREM score | 14.9783 | 16.0909 | 16.6615 | 1.1126 | 7.4% | 1.6832 | 11.2% |

**Table S3.** Sensitivity Analysis: Mean PREM scores Before and After Scale Change

| Dimension | 1st period (2011-2023) | 2nd period (2024-2025) | Absolute change | Relative change |
|---|---|---|---|---|
| Food quality | 1.8015 | 2.1865 | 0.3850 | 21.4% |
| Presentation of meals | 1.9359 | 2.5131 | 0.5744 | 29.7% |
| Cooking of the food | 1.7768 | 2.1399 | 0.3631 | 20.4% |
| Satisfaction with the taste | 1.6638 | 2.2577 | 0.5939 | 35.7% |
| Variety of the diet | 1.5527 | 1.6495 | 0.0968 | 6.2% |
| Food temperature | 1.7917 | 2.3037 | 0.5120 | 28.6% |
| Portion size | 2.0257 | 2.8953 | 0.8696 | 42.9% |
| Timing of meal distribution | 2.3039 | 2.5916 | 0.2877 | 12.5% |
| PREM score | 14.7998 | 18.5397 | 3.7398 | 25.3% |

**Table S4**. Missing Data Analysis by Year

| Year | N | ≥1 missing item (N) | ≥1 missing item (%) |
|---|---|---|---|
| 2011 | 196 | 8 | 4.1% |
| 2012 | 182 | 9 | 4.9% |
| 2013 | 173 | 10 | 5.8% |
| 2014 | 100 | 6 | 6.0% |
| 2015 | 94 | 3 | 3.2% |
| 2016 | 99 | 12 | 12.1% |
| 2017 | 95 | 6 | 6.3% |
| 2018 | 97 | 3 | 3.1% |
| 2019 | 96 | 6 | 6.3% |
| 2020 | 67 | 0 | 0.0% |
| 2021 | 68 | 3 | 4.4% |
| 2022 | 78 | 7 | 9.0% |
| 2023 | 79 | 1 | 1.3% |
| 2024 | 95 | 3 | 3.2% |
| 2025 | 99 | 2 | 2.0% |
| Total | 1618 | 79 | 4.9% |

**Table S5**. Missing Data Analysis by Type of Diet

| Type of diet | N | ≥1 missing item (N) | ≥1 missing item (%) |
|---|---|---|---|
| Regular Diet | 978 | 40 | 4.1% |
| Soft Diet | 265 | 12 | 4.5% |
| Pureed diet | 353 | 25 | 7.1% |
| Soft diet without food that poses a choking risk | 20 | 1 | 5.0% |
| Liquid diet | 1 | 0 | 0.0% |
| NA (type of diet not recorded) | 1 | - | - |
| Total | 1618 | 79 | 4.9% |

**Table S6.** Missing Data Analysis by Hospital Unit

| Hospital Unit | N | ≥1 missing item (N) | ≥1 missing item (%) |
|---|---|---|---|
| Comprehensive Medical Area (CMA) | 930 | 50 | 5.4% |
| Mental Health (MH) | 688 | 29 | 4.2% |
| Total | 1618 | 79 | 4.9% |
